# Localized Chronic Form of Langerhans Cell Histiocytosis in the Femur of a 16-Year-Old Male Successfully Treated with Radiofrequency Ablation

**DOI:** 10.1155/2020/4052034

**Published:** 2020-06-16

**Authors:** Kyriakos Papavasiliou, Antonia Bintoudi, Apostolos Vlahodimos, Eleftherios Tsiridis, Prodromos Hytiroglou, Ioannis Tsitouridis, Fares Sayegh

**Affiliations:** ^1^3^rd^ Orthopaedic Dept., Aristotle University of Thessaloniki Medical School, Papageorgiou General Hospital, Thessaloniki Ring Road West, Nea Efkarpia, 56403 Thessaloniki, Greece; ^2^Department of Radiology, Papageorgiou General Hospital, Thessaloniki Ring Road West, Nea Efkarpia, 56403 Thessaloniki, Greece; ^3^Department of Pathology, Aristotle University of Thessaloniki Medical School, University Campus, 54124 Thessaloniki, Greece

## Abstract

Only three cases of patients suffering from a localized chronic form of Langerhans cell histiocytosis (LCH) successfully treated with radiofrequency ablation (RFA) have been published so far. This is the first case report of a patient with a localized chronic form of LCH of the femur, which was successfully treated with percutaneous image-guided RFA, and who was evaluated pre-RFA and followed up post-RFA for a period of 48 months, in order to validate the safety and efficacy of this method and to obtain imaging studies depicting the actual in situ changes taking place post-RFA. RFA was proved to be a safe and efficient method when treating patients with a localized chronic form of LCH.

## 1. Introduction

Langerhans cell histiocytosis (LCH) is a relatively rare disease of the musculoskeletal system [1, 2]. Being more common in male than female patients, LCH is characterized by the existence of single or multiple skeletal lesions, occurring predominately in patients under twenty years of age and accounts for approximately 70% of the histiocytic neoplasms [3]. The localized chronic form of LCH is an even more rare form of the disease [1, 2]. Several therapeutic methods for the treatment of LCH have been proposed and used with reported results of varying success [4].

The image-guided percutaneous thermoablation is a well-established treatment method for both the primary and the metastatic tumors of the musculoskeletal system. The goal of this procedure is to induce coagulation necrosis in the target tissue, by inserting special applicators in the tumor under image guidance. The lesion is heated to temperatures above 80°C, usually with the use of the radiofrequency ablation (RFA) technique [5–7] [8]. The indications for RFA seem to continuously expand, mainly due to the significant advantages that this method has over surgical treatment [6, 7].

The treatment of the localized chronic form of LCH with the use of RFA was recently introduced in the English literature, and promising results have been reported following its implementation in three young patients [9, 10]. The aim of this study is to present a new case of a patient with a localized chronic form of LCH in his right femur, who was successfully treated with RFA, and to present—to the best of our knowledge—for the first time, long-term imaging studies post-RFA, validating and proving the safety and the efficacy of this novel method for the treatment of the localized chronic form of LCH.

## 2. Case Presentation

**Figure 1:**
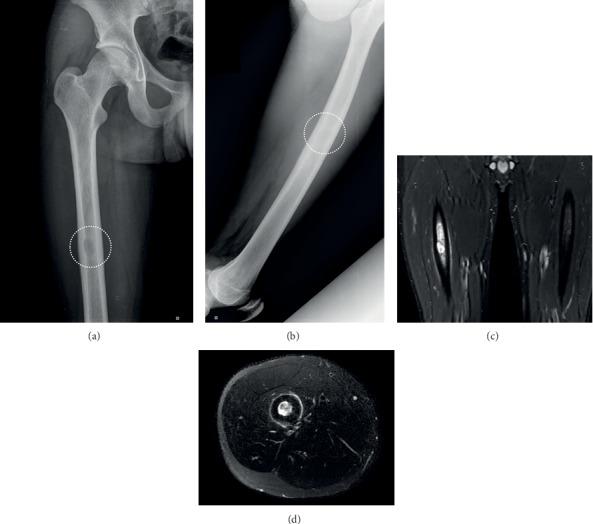
The initial plain radiograph of the femur ((a) anteroposterior and (b) lateral) depicting (dotted line) a centrally located lytic lesion at the femoral diaphysis with concomitant reactive cortical thickness. The initial MRI scan of the lesion. (c) Short T1 reverse- (STIR-) weighted image (WI) coronal view. (d) T2WI axial view (see text for more details).

An otherwise fit 16-year-old boy was referred for evaluation and treatment to our department (a tertiary Trauma and Orthopaedics center) by a general practitioner. The patient was suffering from pain on the lower right thigh with an onset of approximately four months before his initial evaluation. The pain was described as being initially dull and intermittent; later, it became continuous and significantly increased in severity. The pain was elicited on palpation at the affected area and aggravated by weight bearing. The ipsilateral hip and knee joints had a normal range of motion. Red and white blood cell count, C-reactive protein, erythrocyte sedimentation rate, biochemistry, and thyroid and parathyroid hormones levels, were all within normal range. Plain radiographs (Figures 1(a) and 1(b)) revealed the existence of a rounded and well-defined lytic lesion at the right femoral diaphysis with evidence of surrounding mild laminated chronic-type periosteal reaction. The patient subsequently underwent a CT and an MRI scan. CT scan revealed a 13 × 24 mm eccentric lytic lesion with intact femoral cortices and no soft tissue involvement. The same lesion was depicted on the MRI scan (Figures 1(c) and 1(d)) as rounded and well-defined, with intermediate MR sign in the T1 and hyperintensity in the T2-weighted images. The lesion demonstrated intense but relatively homogenous enhancement after the intravenous administration of contrast. On the short T1 reverse- (STIR-) weighted images, intense surrounding bone oedema and a linear periosteal reaction were present.

The patient underwent core needle biopsy under general anesthesia and C-arm X-ray image intensifier control (in order to accurately locate radiographically the exact location of the lesion) with a 6 mm trocar. Culture swabs were also taken from the lesion area and came back as negative. The histopathologic study of the specimen showed a mixed cellular infiltrate made up of macrophages, lymphocytes, eosinophilic granulocytes, multinucleated cells, and Langerhans cells. Further, immunohistochemistry showed positive staining for S-100 protein and CD-1a antigen and confirmed the diagnosis of LCH. Further imaging studies (plain radiographs of the thoracic and lumbar spine, skull, and pelvis) and a Tc^99^ bone scan verified the unifocal form of the disease, and a bone marrow biopsy failed to reveal any other pathology.

Taking into consideration the solitary form of the disease, its location in a weight-bearing long bone, and the age of the patient and following consultation with a consultant radiologist, a pediatric-oncologist, and a pediatric-hematologist, we decided to perform RFA instead of open curettage or wide excision of the lesion followed by bone grafting and/or internal fixation.

The radiofrequency ablation procedure took place in the CT suite room under general anesthesia. A thin biopsy needle was initially introduced through the thickened femoral cortex via the track of the previously performed biopsy. A 15 cm/11-gauge RFA electrode needle (AMICA RF, HS Hospital Services S.p.A., Rome-Italy) was used. The RFA procedure was initiated following confirmation that the tip of the plain electrode was well placed in the center of the lesion. The electrode tip was heated up to 90-94°C for 8 minutes. After a cooling period of 1 minute, the electrode was safely retracted, followed by the probe and the cannula. The patient reported immediate relief from pain as soon as he woke up and was uneventfully discharged the day after. He was advised to walk without bearing weight and with the use of two crutches for one month.

**Figure 2:**
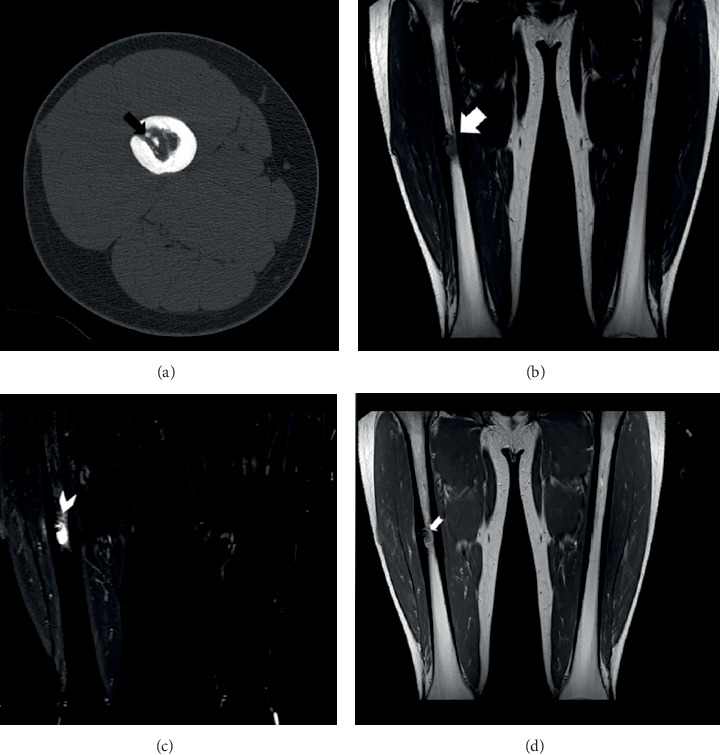
Axial CT image (a) at three months post-RFA, revealing the existence of dystrophic calcifications (black arrow). Coronal MRI scan images of the diaphysis of the right femur of the patient at three months post-RFA, depicting a well-defined lytic lesion with central necrosis. The necrosis had an intermediate to low MR sign on T1WI ((b), white arrow) and prominent surrounding oedema on STIR ((c), arrowhead). After the intravenous administration of contrast media, the lesion showed enhancement only at its periphery ((d) short arrow).

**Figure 3:**
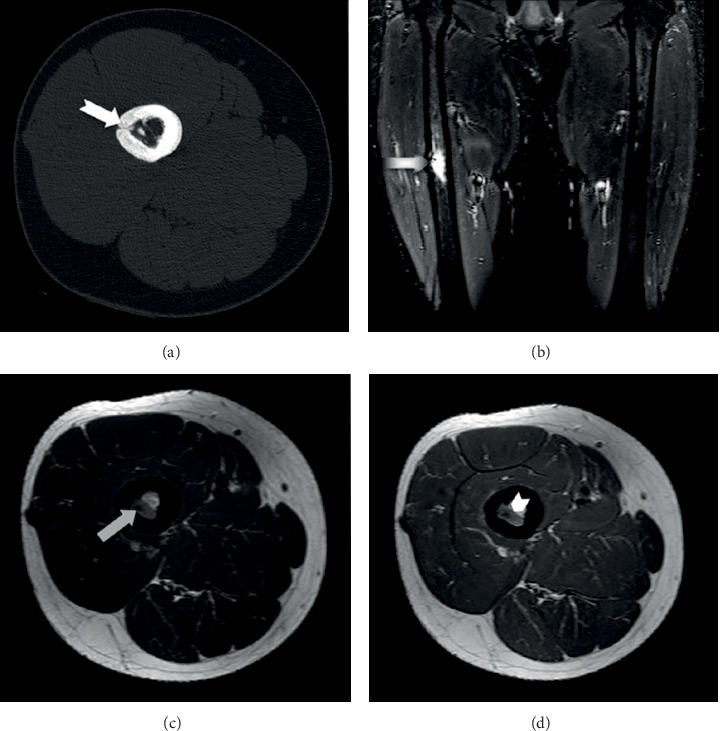
Axial CT scan (a) at six months post-RFA, depicting the formation of callous at the cortex hole area (white arrow). MRI scan at six months post-RFA. (b) Coronal STIR images showing decreased surrounding oedema (when compared with the previous scan) and necrotic appearance of almost the entire lesion (black and white arrow). Axial T1WI images before (c) and after (d) the administration of contrast media, depicting the central necrosis (grey arrow) and the peripheral type of enhancement (short arrow).

**Figure 4:**
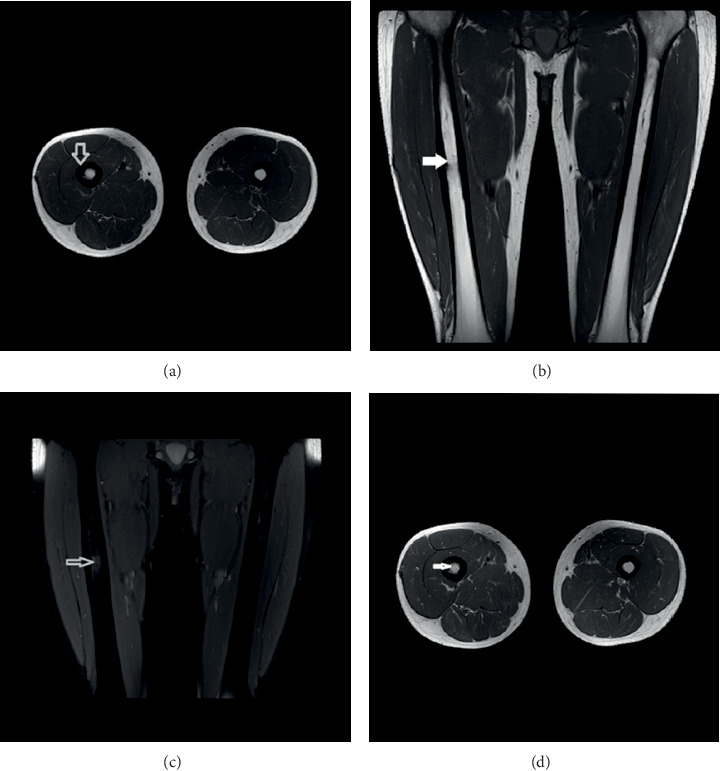
MRI scan at 48 months post-RFA. Axial (a) and coronal (b) T1WI images show no bone oedema (white arrow) and only minor cortical thickening (arrowhead). Proton density WI (PDWI) with fat suppression MRI scan image (c) verified the absence of bone oedema (grey arrow). Axial T1WI MRI scan image (d) following the administration of contrast media suppression show no enhancement.

At three months after the treatment, the patient was completely asymptomatic and able to walk with full weight bearing. The CT scan revealed a central zone with fluid density due to necrosis (Figure 2(a)). The MRI scan confirmed the central necrosis of the tumor, a moderate enhancement at the periphery of the lesion and a mild surrounding bone oedema, which was a consequence of the RFA procedure (Figures 2(b)–2(d)). At six months post-RFA, he reported no pain at all, he was capable of performing all physical activities, and he was completely happy with the overall result of his treatment. The CT scan depicted the existence of callus at the cortex hole area (Figure 3(a)). The MRI scan revealed a more prominent central necrosis, while the post-contrast enhancement and the surrounding bone oedema were decreased (Figures 3(b)–3(d)). During subsequent follow-up visits at 12, 18, 24, and 36 months post-RFA, the MRI characteristics depicting necrosis at the RFA area gradually subsided, until they became normal at the last follow-up visit at month 48 (Figure 4). The patient remains still symptoms-free at his latest phone call follow-up visit six years post-RFA.

## 3. Discussion

The localized chronic form of Langerhans cell histiocytosis of the bone is a benign tumor-like condition, representing less than 1% of the tumor-like lesions of the bone [11, 12]. About 90% of patients are between 5 and 15 years of age and there is a slight male predominance [11]. The bones which are the most commonly involved are the skull and the pelvis [13]. Over one-third of the lesions involve the long bones, with the femur being the most common site, followed by the humerus and tibia. Fibular involvement is rare. In the long bones, the diaphysis is most commonly affected, followed by the metaphysis and rarely the epiphysis [14].

The primary target when treating patients with the localized chronic form of LCH is to achieve complete relief from pain, to prevent pathological fracture and growth disturbances, and to allow returning of the patient to his/her normal everyday-life activities [2, 4, 9]. Several forms of treatment have been proposed, ranging from the simple observation to significantly more invasive ones [13]. The treatment of choice seems to be wide excision and/or curettage, followed by bone grafting [9]. However, operative treatment in particular, although it seems to be rather successful in terms of symptom resolution and low recurrence rates, it may be accompanied by increased morbidity and complications. Alternatively, and in search of a less invasive treatment method, the intralesional instillation of steroids and the external radiation have also been tried, with varying success rates [9]. Complete resolution of the symptoms is reported in 92% and complete imaging resolution in 95% of the patients following the intralesional injection of methylprednisolone [15]. Chemotherapy and external beam radiation have also been reported to have very good results, with a recurrence rate of less than 20% [13]. However, chemotherapy is not easily accepted by a patient for a benign condition and both treatment modalities may lead to the later development of severe complications.

Radiofrequency ablation under radiographic guidance is a well-established and easy-to-perform method of treatment for several benign and malignant bone lesions [5]. This method offers immediate pain relief, shorter rehabilitation period for the patient, brief hospitalization, less cost, less soft tissue damage, and minimal blood loss [16, 17]. Due to all these advantages and the fact that its use does not necessitate the purchase of expensive equipment, it has gained worldwide acceptance and recognition. Its use in the treatment of patients suffering from LCH has been recently introduced and the results reported so far in three patients are very promising [9, 10].

We report—to the best of our knowledge—the fourth case in the English literature of a patient suffering from a localized chronic form of LCH who was successfully treated with RFA (Table 1). The patient experienced immediate relief from his symptoms, suggesting adequate and successful treatment. Furthermore, the imaging studies obtained following the RFA prove beyond any doubt the therapeutic value of this novel method of treatment, by clearly depicting the complete necrosis of the lesional area and the subsequent regeneration of normal tissue during the following 48 months.

Even though this is a case report, there are some limitations that must be mentioned. One is that the patient could have been successfully treated with alternative therapeutic methods as well, even with a simple observation. However, he refused to do so because he could not tolerate the pain and he wanted a permanent and rapid treatment for his condition. PET CT, which is also a valuable modality in the primary assessment, therapeutic monitoring, and detection of reactivation of patients with LCH , could also have been used as a means to evaluate the efficacy of the RFA procedure during the follow-up evaluations. However, the increased cost and the dosimetric considerations for ionizing radiation uptake were the reasons we chose the MRI scans instead.

Radiofrequency ablation, as a novel method of treatment for the localized chronic form of LCH, certainly needs to be further validated by a large series of patients. Given however the rarity of the disease, the previously reported and effectively treated three patients, the hereby reported patient, and the reduced morbidity rates accompanying this method, it seems that RFA is a highly successful method of treatment when dealing with patients suffering from a localized chronic form of Langerhans cell histiocytosis.

## Figures and Tables

**Table 1:** Summary of the previously reported patients' characteristics and outcomes and comparison with this study's data. M: male, F: female, RFA: radiofrequency ablation.

Study	Age	Sex	Location of the lesion	Outcome	Latest follow-up	Imaging modality used
Corby et al.	7	F	Femoral diaphysis	Full resolution of symptoms 7 days post-RFA	12 months post-RFA	Radiograph
	14	F	Iliac	Full resolution of symptoms 2 days post-RFA	11 months post-RFA	Radiograph
Beccee et al.	18	M	Iliac	Full resolution of symptoms	12 months post-RFA	N/A
Present study	16	M	Femoral diaphysis	Full resolution of symptoms immediately post-RFA	48 months post-RFA	Radiograph, CT scan, MRI scan
